# To Remove Teeth from Rubber Plate without Danger of Cracking or Etching the Teeth

**Published:** 1900-03-15

**Authors:** 


To Remove Teeth from Rubber Plate Without Danger of Cracking or Etching the Teeth.—Boil the plate in glycerin, in a porcelain pan, till it smokes, and the teeth will come away clean and free from discoloration. Put them back in the glycerin to anneal them, and when cool wash in warm water. They will be as bright as when new. The glycerin can be bottled for future use.—*Dr. Genese, Ohio Dental Journal.*

